# Potential Pro-Inflammatory Effect of Vitamin E Analogs through Mitigation of Tetrahydrocannabinol (THC) Binding to the Cannabinoid 2 Receptor

**DOI:** 10.3390/ijms23084291

**Published:** 2022-04-13

**Authors:** Anjela Manandhar, Mona H. Haron, Samir A. Ross, Michael L. Klein, Khaled M. Elokely

**Affiliations:** 1 Institute for Computational Molecular Science and Department of Chemistry, Temple University, Philadelphia, PA 19122, USA; anjela.manandhar@temple.edu (A.M.); mlklein@temple.edu (M.L.K.); 2 National Center for Natural Products Research, University of Mississippi, University, MS 38677, USA; sross@olemiss.edu; 3 Department of BioMolecular Sciences, School of Pharmacy, University of Mississippi, University, MS 38677, USA

**Keywords:** EVALI, THC, CB2R, MD simulation, inflammatory

## Abstract

Vitamin E acetate, which is used as a diluent of tetrahydrocannabinol (THC), has been reported as the primary causative agent of e-cigarette, or vaping, product use-associated lung injury (EVALI). Here, we employ in vitro assays, docking, and molecular dynamics (MD) computer simulations to investigate the interaction of vitamin E with the membrane-bound cannabinoid 2 receptor (CB2R), and its role in modulating the binding affinity of THC to CB2R. From the MD simulations, we determined that vitamin E interacts with both CB2R and membrane phospholipids. Notably, the synchronized effect of these interactions likely facilitates vitamin E acting as a lipid modulator for the cannabinoid system. Furthermore, MD simulation and trajectory analysis show that when THC binds to CB2R in the presence of vitamin E, the binding cavity widens, facilitating the entry of water molecules into it, leading to a reduced interaction of THC with CB2R. Additionally, the interaction between THC and vitamin E in solution is stabilized by several H bonds, which can directly limit the interaction of free THCs with CB2R. Overall, both the MD simulations and the in vitro dissociation assay results indicate that THC binding to CB2R is reduced in the presence of vitamin E. Our study discusses the role of vitamin E in limiting the effect of THCs and its implications on the reported pathology of EVALI.

## 1. Introduction

In 2019, the United States was grappling with an outbreak of flu-like illness named e-cigarette, or vaping, product use-associated lung injury (EVALI) [1,2]. The patients were a mostly younger population with a history of using tetrahydrocannabinol (THC)-containing e-cigarettes, or vaping products within three months prior to the onset of symptoms. As of February 2020, a total of 2807 hospitalized EVALI cases with 68 deaths have been reported in the USA [1]. A study by the Lung Injury Response Laboratory Working Group [3] reported the presence of vitamin E acetate in bronchoalveolar-lavage fluid in 48 of the 51 EVALI patients. Thus, vitamin E acetate has been suggested as the prime toxic agent for EVALI.

Vitamin E acetate with ester moiety is thermostable and lacks the antioxidant property of vitamin E [4]. Thus, the acetate form is used as the dietary supplement of vitamin E that is readily hydrolyzed by cellular esterase as carboxyl ester hydrolase and cholesteryl ester hydrolase [5,6]. However, vitamin E acetate is hazardous when inhaled as vapor as it does not undergo esterase-mediated hydrolysis [7]. The CDC has reported that a sticky, honey-like vitamin E acetate can hang around in the lungs for several hours, affecting pulmonary functions [8].

Vitamin E is a major lipid-soluble component in the cell antioxidant defense system [9]. Because of its antioxidant property, it has been found to be effective in the prevention and reversal of various health conditions including cancer, cataract, cardiovascular disease, and Alzheimer’s disease [9]. Although vitamin E’s antioxidant property is well recognized, its non-antioxidant property is slowly gaining interest. Vitamin E’s role in inhibiting protein kinase C, cell proliferation, and transcription of some genes (for example CD36 and collagenase) have been attributed to its non-antioxidant property [10]. Crouzin et al. [11] in their study of rodent hippocampus suggested vitamin E as the new lipid modulator of the cannabinoid system in the rodent hippocampus. It has been suggested that the novel “non-anti-radical” property of vitamin E might affect neuronal disorders associated with vitamin E deficiency.

**Figure 1:**
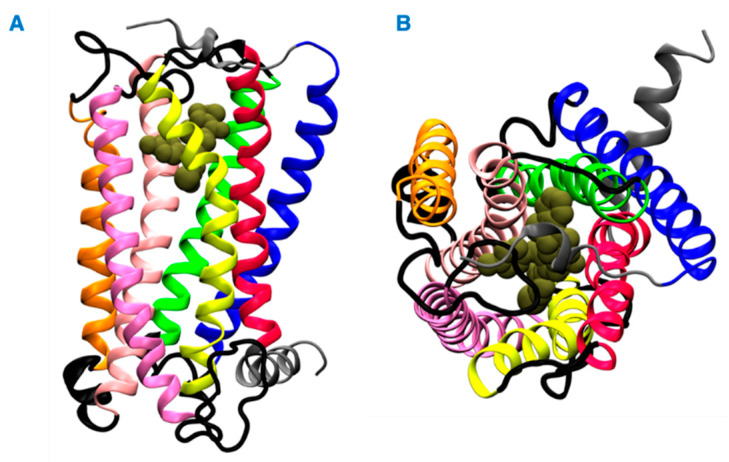
Structure of the CB2 receptor. (**A**) Side view. (**B**) Top view. The transmembrane helices 1, 2, 3, 4, 5, 6, and 7 are shown in blue, green, light pink, orange, pink, yellow and red colors, respectively. The intracellular and extracellular loops are shown in black, and the extracellular N terminus, helix 8, and the intracellular C terminus are shown in gray. A representative ligand in the orthosteric binding site is rendered in tan color spheres.

**Figure 2:**
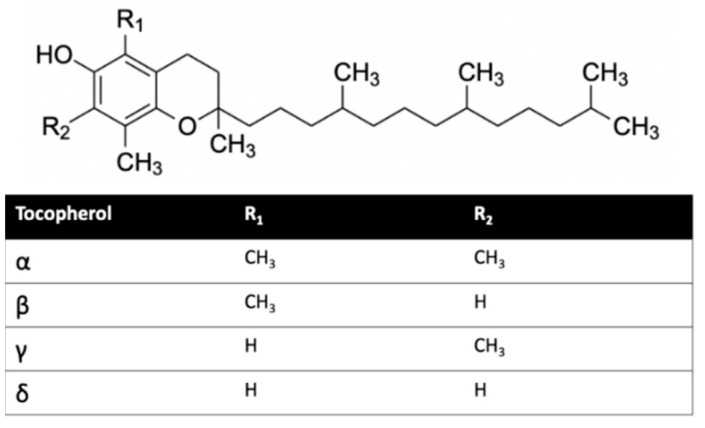
Vitamin E structures.

Herein, we investigated the interaction of vitamin E with cannabinoid 2 receptor (CB2R) and studied the behavior of vitamin E and its effect on THC binding. CB2R is one of the two human cannabinoid receptors and belongs to class A of the G-protein coupled receptor (GPCR) family. CB2R is primarily distributed in the immune system [12,13]. CB2R has been reported to have a potential role in regulating pain, pruritus, neuropathy, and liver cirrhosis [14,15,16,17,18,19,20,21]. The first crystal structure of human CB2R in complex with the antagonist AM10257 was reported in 2019. Like other GPCRs, CB2R consists of seven transmembrane (TM) helices (H 1 to 7) and one intracellular amphipathic helix (H8) (Figure 1). Residues of each transmembrane helices is shown in Table S1. These helices are connected by three extracellular loops (EL 1 to 3) and three intracellular loops (IL 1 to 3). Figure 1 shows a ligand binding at the orthosteric binding cavity. The cavity is located near the extracellular region and is surrounded by H2, H4, H5, H6, and H7. Vitamin E structures are differentiated from one another by the number and position of the methyl groups on the aromatic ring (Figure 2). For this study, we performed molecular dynamics (MD) simulations of CB2R with each type of vitamin Es (α, β, γ, and δ) organized around it, and CB2R with THC at its orthosteric binding site in the presence and absence of α vitamin Es. The study suggests CB2R has weaker interaction with vitamin Es compared to phospholipids with vitamin Es. THC has reduced interactions with CB2R in the presence of α vitamin E with a wider binding cavity and an increased number of water molecules in the cavity. Besides the interactions of CB2R and vitamin Es, we found the strong interaction between THCs and α vitamin Es could also limit the interaction of THCs to CB2R.

## 2. Results and Discussion

### 2.1. Effect of Vitamin E Acetate on THC-CB2 Binding Affinity In Vitro

**Figure 3:**
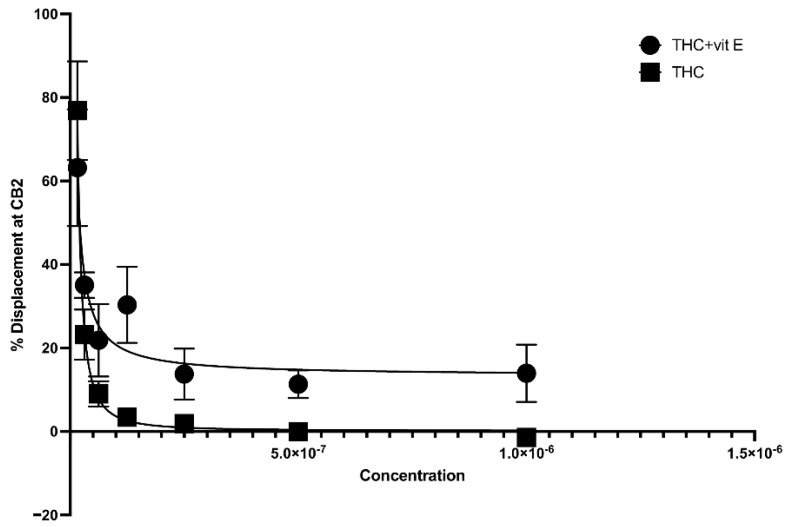
Binding affinity of THC to CB2R with and without vitamin E acetate.

The concentration of vitamin E acetate in bronchoalveolar-lavage fluid samples, as reported by the CDC, ranges from 23% to 88% [2]. In this study, we explored the possibility that the presence of vitamin E acetate in e-cigarettes, and vaping THC products, affects the binding affinity of THC to CB2R. A 50% vitamin E acetate to 50% THC in propylene glycol (in different concentrations) volume wise in comparison to 50% vegetable glycerin (a commonly used e-cigarette/vape diluent) 50% THC in propylene glycol (in different concentrations) were used to test the affinity of THC for CB2R by using a displacement assay. THC-CB2R binding properties were investigated by applying a classical radioactivity-based assay wherein a conventional radioligand [3H] CP-55,940, that has low affinities toward CB2Rs was utilized. THC binding affinity to CB2R is calculated by its ability to displace [3H] CP-55,940. Our data showed that in the presence of vitamin E acetate, THC is less able to displace [3H] CP-55,940 and bind to CB2R at concentrations ranging from 31.25–250 µg/mL by up to 20% (EC50 = 4.97 µg/mL) in comparison to THC without vitamin E acetate (EC50 = 5.62 µg/mL) with *n* = 3/assay. No significant displacement was observed at the lower concentrations in the presence of vitamin E acetate (Figure 3). This data suggest that vitamin E acetate could decrease THC binding to CB2R and possibly decrease THC-CBR2 mediated anti-inflammatory activity. The decreased THC binding to CB2R could be due to the formed THC–vitamin E acetate adduct which decreases the free THC that could bind to CB2R.

### 2.2. Interaction of Vitamin Es with CB2R

**Figure 4:**
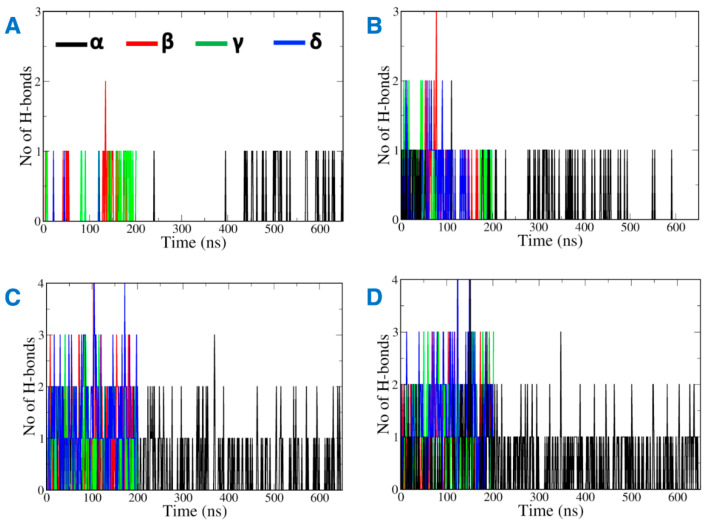
Hydrogen bonds between vitamin Es and (**A**) the active state structure of CB2R, (**B**) the inactive state structure of CB2R, (**C**) the phospholipid bilayer surrounding the active state structure of CB2R, and (**D**) the phospholipid bilayer surrounding the inactive state structure of CB2R.

Most of the vitamin E molecules organized around CB2R distributed in the solvent and remained close to the phospholipid bilayer (Figure S1). The analysis of MD trajectories revealed that the hydrophobic vitamin Es have greater inclination to interact with the phospholipid bilayer of the membrane than with CB2R. Hydrogen bonds contribute favorably towards the stabilization of ligand binding to its biological target. Interestingly, the number of hydrogen bonds monitored throughout the MD simulations between vitamin E and the phospholipid was found to be greater than the hydrogen bonds formed between vitamin E and CB2R (Figure 4). While, the hydrogen bonds with CB2R fluctuated during the MD simulations (Figure 4A,B), they were consistent with phospholipids (Figure 4C,D). The hydrogen bonds were lost between α vitamin Es and the active state structure of CB2R from 200 ns to 450 ns of the MD simulations (Figure 4A), and lost throughout 200 ns to 300 ns, and 500 ns to 600 ns in the case of the inactive state (Figure 4B).

**Figure 5:**
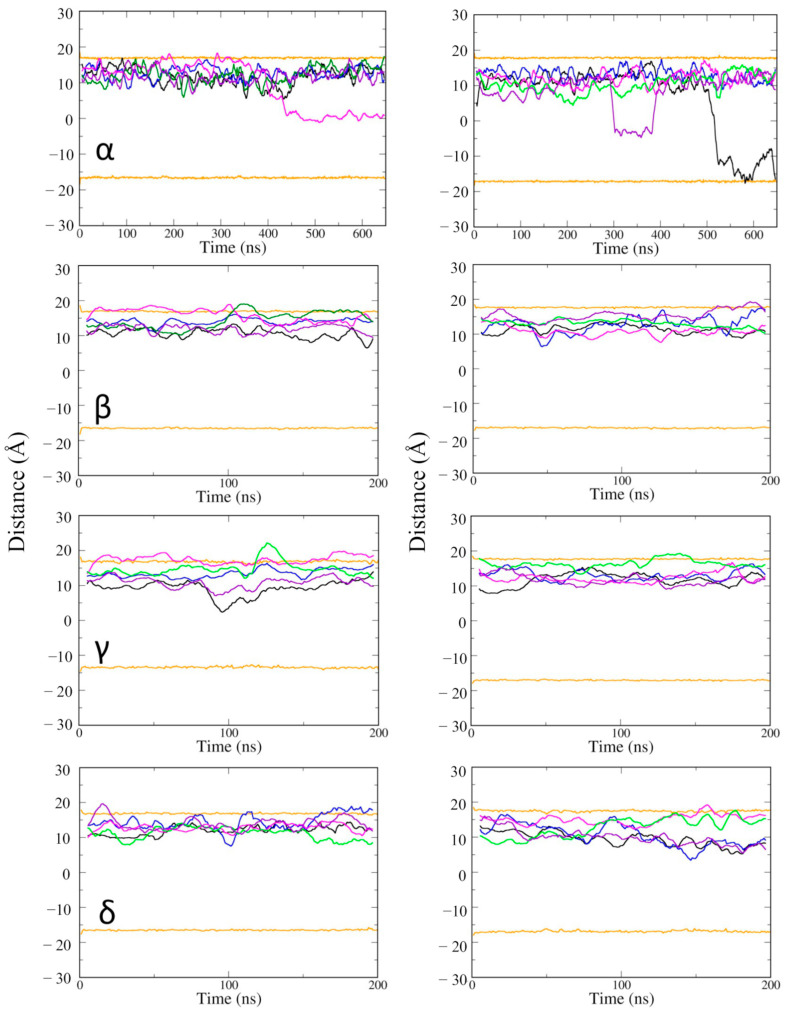
The average positions of vitamin Es in the membrane of active state (**left panel**) and inactive state structures (**right panel**) of CB2R. The average positions of phosphates in the upper and lower layers are shown as orange lines, and the positions of the five vitamin Es as black, blue, magenta, green, and violet lines.

Next, the position of vitamin Es was investigated along the depth of the phospholipid bilayer membrane (Figure 5). Some of the vitamin E molecules moved across the membrane from the upper to the lower leaflet. The movement was not specific to the type of vitamin E, but it took place at a long time scale, after about 300 ns. It suggested that if vitamin Es can access and interact with the lower POPC layer, they can travel from the upper leaflet to the lower leaflet of the cell membrane. Vitamin Es fluctuated within 10 Å of the upper membrane leaflet during the 200 ns MD simulations. γ vitamin E moved to the upper edge of the lower leaflet at 100 ns. The MD simulations of the α vitamin E system was extended to 650 ns to have a better understanding for the membrane permeability. α vitamin E moved to the upper edge of the lower leaflet of the active state structure of CB2R at 450 ns, and it moved deeper into the lower leaflet of the inactive state starting from ~300 ns, and almost penetrated the membrane at the end of simulation.

**Figure 6:**
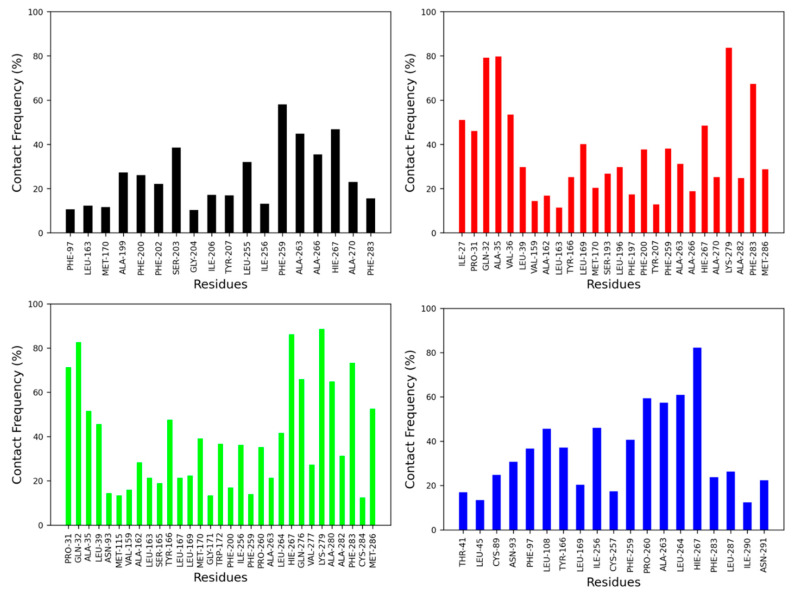
Contact frequencies of the amino acid residues of the active state structure of CB2R with α (black), β (red), γ (green) and δ (blue) vitamin Es.

**Figure 7:**
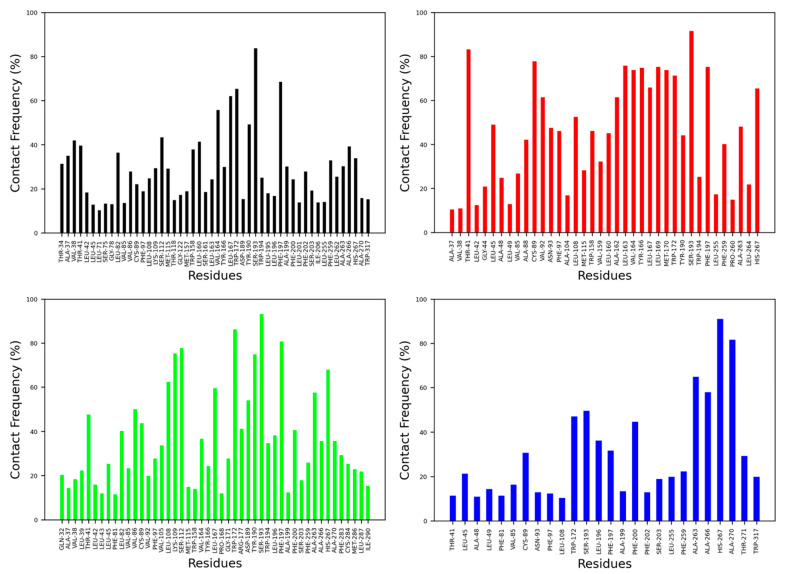
Contact frequencies of the amino acid residues of the inactive state structure of CB2R with α (black), β (red), γ (green) and δ (blue) vitamin Es.

The contact frequency was monitored between CB2R amino acid residues and vitamin Es throughout the MD simulations (Figure 6 and Figure 7). For the active state structure of CB2R, H6 showed the most contacts with α and δ vitamin Es, whereas H1 and H7 were found to interact more with β, γ, and δ vitamin Es. The CB2R residues that showed interactions more than 40% of the simulation time with α vitamin E include Ser^203^, Phe^259^, Ala^263^, Ala^266^ and His^267^, whereas with β vitamin E the residues are Ile^27^, Pro^31^, Gln^32^, Ala^35^, Val^36^, Leu^169^, His^267^, Lys^279^ and Phe^283^, with γ vitamin E residues including Pro^31^, Gln^32^, Ala^35^, Leu^39^, Tyr^166^, Leu^264^, His^267^, Gln^276^, Lys^279^, Ala^280^, Phe^283^ and Met^286^, and in the case of δ vitamin E, the residues include Leu^108^, Ile^256^, Phe^259^, Pro^260^, Ala^263^, Leu^263^, Leu^264^ and His^267^. Here, His^267^ of H6 was the only common residue with contact frequency of more than 40% with all vitamin Es. For the inactive state structure of CB2R, H5, and H6 showed prominent contacts with all types of vitamin Es, whereas H4 and H5 were involved in more contacts with α and β vitamin Es, H1 with β and γ vitamin Es, H2 with β vitamin E, and H3 with γ vitamin E. The CB2R residues that showed contacts over 40% of the simulation time with α vitamin E include Val^38^, Thr^41^, Ser^112^, Trp^158^, Leu^160^, Val^164^, Leu^167^, Trp^172,^ Tyr^190^, Ser^193^, Phe^197^, Ala^266^, and His^267^. For β vitamin E the list includes Val^41^, Leu^45^, Ala^88^, Cys^89^, Val^92^, Asn^93^, Phe^97^, Leu^108^, Trp^158^, Leu^160^, Ala^162^, Leu^163^, Val^164^, Tyr^166^, Leu^169^, Met^170^, Trp^172^, Tyr^190^, Ser^193^, Phe^197^, Phe^259^, Ala^263^ and His^267^, and for γ vitamin E the residues are Thr^41^, Leu82, Val^86^, Cys^89^, Leu^108^, Lys^109^, Ser^112^, Leu^167^, Trp^172^, Arg^177^, Asp^189^, Tyr^190^, Ser^193^, Phe^197^, Phe^200^, Ala^263^, and His^267^. With δ vitamin E, the residues include Trp^172^, Ser^193^, Phe^200^, Ala^263^, Ala^266^, His^267^ and Ala^270^. Trp^172^, Ser^193^, and His^267^ were the common residues with contact frequency greater than 40% with all vitamin Es. In general, vitamin Es interact with different helices of CB2R which may affect the THC binding with CB2R.

### 2.3. Effect of Vitamin Es on THC Binding to CB2R

**Figure 8:**
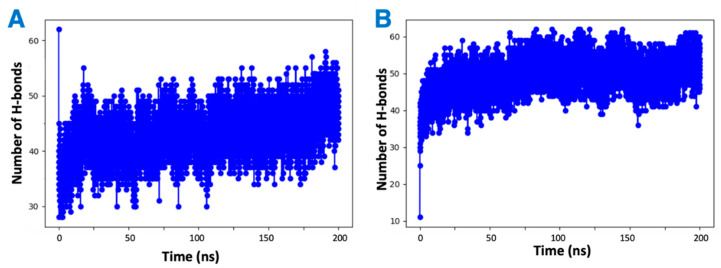
Changing number of hydrogen bonds in the mixtures of hydrogen-bonded THC and α vitamin Es in implicit solvent over 200 ns simulation. (**A**) THC is the hydrogen bond donor. (**B**) THC is the hydrogen bond receiver.

It was observed by in vitro studies that the presence of vitamin Es leads to reduced THC binding to CB2R. To understand this phenomenon, four MD simulations were attempted: first a mixture of THC and α vitamin E in implicit solvent to test the possibility of aggregate (adduct) formation, the other three systems for the active state structure of CB2R in complex with THC in the absence and presence of α vitamin Es or its acetate to test the direct effect of vitamin E on ligand binding. Analysis of the MD simulations of the equal (1:1) mixture of α vitamin E and THC reveals that THC could participate in the formation of hydrogen bonds with α vitamin E with a hydrogen bond donor or receptor group (Figure S2). These hydrogen bonded systems were replicated 64 times for MD simulations in implicit solvent for 200 ns. The total number of hydrogen bonds in each system was monitored over the simulation time. As shown in Figure 8, each system had more than forty hydrogen bonds throughout the simulation, suggesting favorable contacts between THC and α vitamin E that may lead to an adduct formation. This stable adducts may limit the availability of free THCs for CB2R binding.

**Figure 9:**
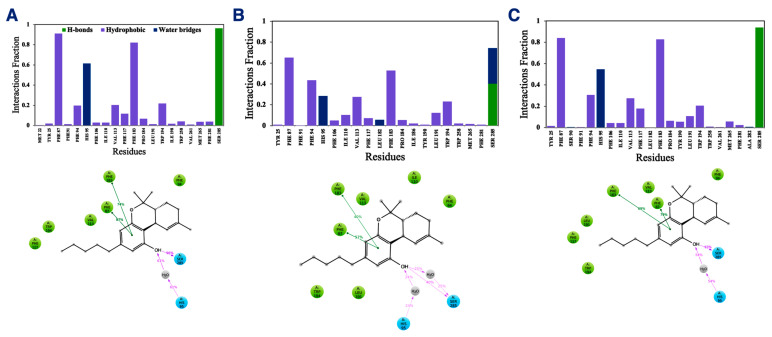
Interaction fractions (upper panel) and 2D interaction diagram (lower panel) of THC with active state structure of CB2R in (**A**) the absence of α vitamin E and its acetate, (**B**) in the presence of α vitamin Es, and (**C**) in the presence of α vitamin E acetate.

**Figure 10:**
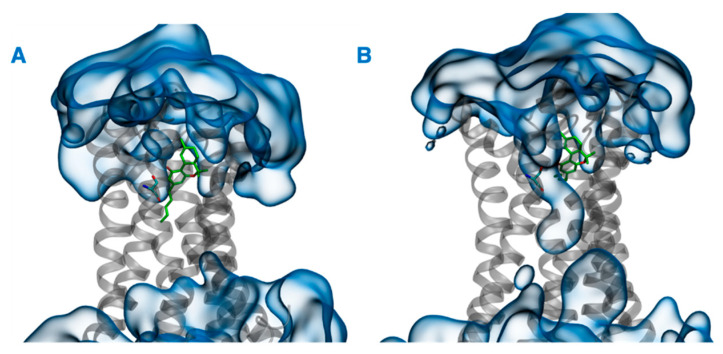
Last frames of (**A**) system 1 and (**B**) system 2. THC and Ser^285^ are shown in green, CB2R in gray, and water in blue.

In addition to the direct effect of vitamin Es on THC binding to CB2R through aggregate formation in the cytosol, the indirect effect was explored by studying the influence of vitamin Es on the ligand binding cavity of CB2R. The active state structure of CB2R in complex with THC at the orthosteric binding site was simulated in the presence and absence of α vitamin E and α vitamin E acetate for 200 ns. Although the interaction of CB2R with THC is affected significantly in the presence of α vitamin Es, the interaction is alike in presence of α vitamin E acetate. (Figure 9). In the case of system 1 (without α vitamin E and its acetate) and system 3 (with α vitamin E acetate), THC showed a strong hydrogen bond with Ser^285^, π-π stacking with Phe^87^ and Phe^183^, and a water-mediated interaction with His^95^. These interactions were consistent throughout the 200-ns MD simulation as shown in Figure S3 and S4. However, in case of system 2 (with α vitamin E), the interactions between Ser^285^, Phe^87^, Phe^183^, and His^95^ with THC demonstrated significant reduction. Moreover, Ser^285^ lost the strong hydrogen bond with THC and rather a water-mediated interaction was developed. Following this, system 1 and system 2 were further investigated. The last frames of system 1 and system 2 were compared as shown in Figure S5. The orientations of THC and Ser^285^ were shifted in system 2 when compared to system 1. Interestingly, by the end of the simulation in case of system 2, H6 protruded outward in the extracellular region (Figure S5), and water molecules entered the binding cavity (Figure 10). Thus, we can conclude that in the presence of α vitamin E, water molecules were attracted to the ligand binding cavity, leading to a new opening and a new interaction pattern.

**Figure 11:**
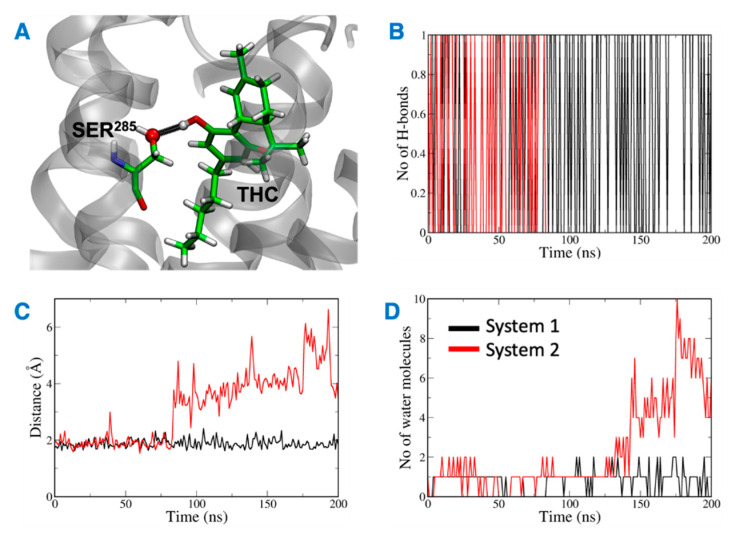
(**A**) Ser^285^ and THC highlighted in green with hydrogen bond between them shown in black. (**B**) Number of hydrogen bonds between Ser^285^ and THC. (**C**) Distance between Ser^285^ and THC hydrogen bond forming atoms. (**D**) Number of water molecules around Ser^285^.

**Figure 12:**
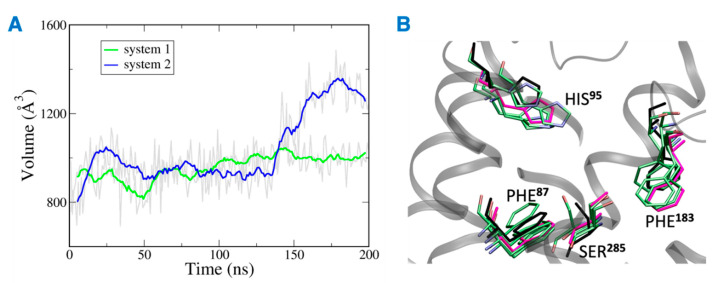
(**A**) Volume of the binding site over 200 ns simulation (gray), running averages shown in green (system 1) and blue (system 2). (**B**) Orientations of residues of system 2 with reduced interactions with THC. Here magenta, black and green represent the initial state, final state, and clusters, respectively.

The distance between the hydrogen bond donor, oxygen from Ser^285^ and acceptor, hydrogen from THC was analyzed over the simulation time (Figure 11). In the case of system 2, the hydrogen bond was lost at ~75 ns, and the donor–acceptor distance increased to more than 3 Å. Whereas, in case of system 1, the hydrogen bond was consistent throughout the 200 ns, and the donor–acceptor distance was maintained within 2 Å. Next, the number of water molecules within 8 Å of Ser^285^ was calculated over the simulation time, and it was found to increase to more than 4 molecules after about 150 ns. This suggests that water molecules moved into the binding cavity after the hydrogen bond was broken. Next, the volume of the binding cavity was calculated for the simulation trajectories by using Fpocket (Figure 12A) [22]. For system 2, the volume of the ligand cavity increased to more than 1200 Å^3^ at about 140 ns, allowing for water molecules to enter the cavity. The orientations of the amino acid residues, Phe^87^, His^95^, Phe^183^, and Ser^285^, which showed the reduced interaction patterns with THC, were compared for the clustered trajectory of system 2 (green color) with the initial frame (magenta color), and final frame (black color) of the CB2R (Figure 12B). As shown in Figure 12B, these residues have shifted orientations. His^95^ and Phe^87^ moved upward, the Ser^285^ side chain moved inward to the binding cavity, and the aromatic ring of Phe^183^ oriented itself perpendicular to its original orientation at the later stage of simulation.

## 3. Materials and Methods

### 3.1. Chemicals

Δ^9^-THC (Neat) was obtained from Coy Waller Laboratory, National Center for Natural Products Research, University of Mississippi. Propylene glycol (99.7%), vegetable glycerin (99.7%), and vitamin E acetate (96–102%) were obtained from EC Blend Premium Artisan Flavor.

### 3.2. CB2 In Vitro Binding Assay

The affinities of THC for CB2R were examined by using displacement assays, as previously described [23]. Briefly, cell membranes from CHO cells expressing human CB2Rs were isolated by using differential centrifugation. THC in PG with and without vitamin E were incubated with the isolated membrane in binding buffer (50 mM Tris-HCl, 1 mM EDTA, 3 mM MgCl2, 5 mg/mL BSA, pH 7.4) along with 2.5 nM [3H] CP-55,940. The total binding was assessed in the presence of equal concentrations of DMSO, whereas nonspecific binding was determined in the presence of 10 μM [3H] CP-55,940, and background binding was determined in wells lacking a membrane. Following incubation at 30 °C for 60 min, the binding reactions is terminated by filtration through Whatman GF/C filters. The filters then are washed twice with an ice-cold buffer (50 mM Tris-HCl, 1 mg/mL BSA). A liquid scintillation cocktail was added to each well and the total tritiated counts per minute were analyzed by using a TopCount scintillation counter. Background counts were subtracted from all wells and the percent displacement from total binding was calculated. THC was screened at 4–250 μg/mL of PG concentrations alone or in the presence of 50% vitamin E acetate. GraphPad Prism 9.3.1 (350) was used to calculate the EC50.

### 3.3. System Setup for MD Simulation

The crystal structure of inactive state CB2R (PDB 5ZTY) was obtained from the protein databank [24]. The active state CB2R was modeled by using the active state CB1R [25] as the template. To facilitate crystallization all structures had been modified with mutations and fused with a stabilizing protein in ICL3. First, these mutations were transformed back to wild-type and fusion proteins were removed. Then, the missing segment of ICL3 was reconstructed by crosslinking the two ends of ICL3 by using the BioLuminate model of the Schrödinger software suite [26,27,28,29]. Next, the CB2R structures were prepared by using the protein preparation wizard workflow of the Schrödinger suite. For the membrane setup, the system builder module of the Desmond code was used [30,31]. All CB2R structures were embedded in the POPC lipid bilayer, neutralized with Na^+^ and Cl^−^ ions, and solvated by TIP3P [32] water. The positions of the CB2R structures in the membrane were based on the CB2R inactive structure in the membrane from OPM database [33]. Next, a layer of vitamin Es was added, with five vitamin E molecules around CB2R. The layer had a diameter of ~40 Å and each vitamin E was inclined at 72° with respect to the other vitamin Es. The layer was positioned such that the -OH group of vitamin Es were at the same level as that of phosphate of the upper POPC leaflet. In total, eight systems were prepared with α, β, γ, and δ vitamin Es around active and inactive CB2Rs (Table 1).

### 3.4. Docking of THC

The binding site for active-state CB2R was prepared by using Glide [34,35,36] along with the atomic coordinates of the bound agonist AM841 in the active state structure of CB1R [25]. THC was prepared for docking by using LigPrep [37] to generate appropriate tautomers and stereoisomers at pH 7.0. Next, THC was docked to active-state CB2R by using the SP (standard precision) algorithm. For more receptor flexibility, the receptor potential was softened by scaling the per-atom van der Waals radii and charges to 0.85 and 0.15, respectively. Then, the pose with lowest docking score was selected for further analysis. Next, the active state CB2R, with docked THC, was prepared with and without α vitamin Es and its acetate surrounding CB2R (Table 1). The same procedure was employed as for the 8 systems described above.

### 3.5. MD Simulations

MD simulations in the NPT ensemble were performed for all 11 systems (Table 1) by using the Desmond code [30,31] of the Schrödinger suite and the OPLS3e [38] force field. The pressure and temperature were kept constant at 1 bar and 300 K respectively, by using the Nosé-Hoover chain and Martyna-Tobias-Klein coupling schemes, respectively [39,40]. For the numerical integration, the RESPA integrator was employed with a short range/bonded interaction and long-range/non-bonded interactions updated every 2 ps and 6 ps, respectively [41]. The short-range Coulomb interactions employed a cutoff of 9.0 Å [42]. The long-range interactions were calculated by using the particle mesh Ewald method with a tolerance of 1 × 10^−9^. Images were generated by using VMD visualization tools [43].

### 3.6. THC and α Vitamin E

THC and α vitamin E were prepared with LigPrep [37] as described before. A mixture of 64 molecules each was placed to have hydrogen bonds between the two molecules. The first mixture was placed in a box with the dimensions of 70.0633 Å × 66.1411 Å × 77.067 Å, and the second box has the following dimensions 70.1718 Å × 76.9387 Å × 74.2976 Å. The simulation protocol was carried out in the same way as described above.

## 4. Conclusions

Herein, we have performed MD simulations of 11 systems of CB2R and vitamin E and 2 systems of THC and vitamin Es to study the direct and indirect effects of vitamin E and its acetate on THC binding to CB2R. All vitamin E subtypes—α, β, γ, and δ interact with CB2R helices. Although the interaction of vitamin E with CB2R is weaker compared to that of vitamin E with phospholipids, the synchronized effect may facilitate vitamin E to act as lipid modulator for cannabinoid systems as hypothesized by Crouzin et al. [11]. In the THC-α vitamin E mixture, there were several stable H-bonds indicating vitamin E can limit the availability of free THCs, thereby reducing THC binding to CB2R. Moreover, in the presence of vitamin E, the binding cavity of CB2R is wider, with the increased access to water molecules resulting in reduced interaction of THC with CB2R. The simulation results of vitamin E agree well with the in vitro data showing reduced THC binding to CB2R in the presence of vitamin E acetate. Based on the simulation analysis of vitamin E and its acetate, it is more likely that the vitamin E acetate hydrolyses to vitamin E as observed in a physiological environment.

Besides having a psychoactive effect, THC also has anti-inflammatory effect through CB2R [44]. Moreover, THC has anti-oxidant activity independent of CB2R [45,46]. The EVALI patients have shown a pathological picture indicative of lung inflammation with unclear etiology. Our study shows that vitamin E could be restricting the ability of THC binding to CB2R. The restricted binding of THC by vitamin E might be a factor leading to downregulation of the anti-inflammatory and anti-oxidative responses of THCs leading to lung inflammation with chronic exposure. Overall, more research is needed to further understand the role of vitamin E/acetate in EVALI outbreaks.

## Figures and Tables

**Table 1:** Different systems and time scales for MD simulation.

Systems			Simulation Time (ns)
		Vitamin E	
CB2R	Active	α	650
		β	200
		γ	200
		δ	200
	Inactive	α	650
		β	200
		γ	200
		δ	200
CB2R + THC		α	200
CB2R + THC		-	200
CB2R + THC		α acetate	200

## Data Availability

Initial .cms files of MD simulations of the 11 systems discussed in Table 1 can be made available on request from the corresponding authors.

## Supplementary Materials

The following supporting information can be downloaded at: https://www.mdpi.com/article/10.3390/ijms23084291/s1.

## Abbreviations

EVALI	E-cigarette, or Vaping, product Use–Associated Lung Injury
THC	Tetrahydrocannabinol
MD	molecular dynamics
CB2R	cannabinoid 2 receptor
