# Supplementary material for: Potential Pro-Inflammatory Effect of Vitamin E Analogs through Mitigation of Tetrahydrocannabinol (THC) Binding to the Cannabinoid 2 Receptor
Source: Int J Mol Sci. 2022 Apr 13;23(8):4291. doi: 10.3390/ijms23084291 (PMC9026873; doi:10.3390/ijms23084291)
Supplement: Supplementary file 1 [file ijms-23-04291-s001.zip › ijms-1667652-supplementary.pdf]

International Journal of
Molecular Sciences

MDPI

Potential Pro-Inflammatory Effect of Vitamin E Analogs Through Mitigation of Tetrahydrocannabinol (THC) Binding to the Cannabinoid 2 Receptor

Anjela Manandhar, Mona H. Haron, Samir A. Ross, Michael L. Klein and Khaled M. Elokely

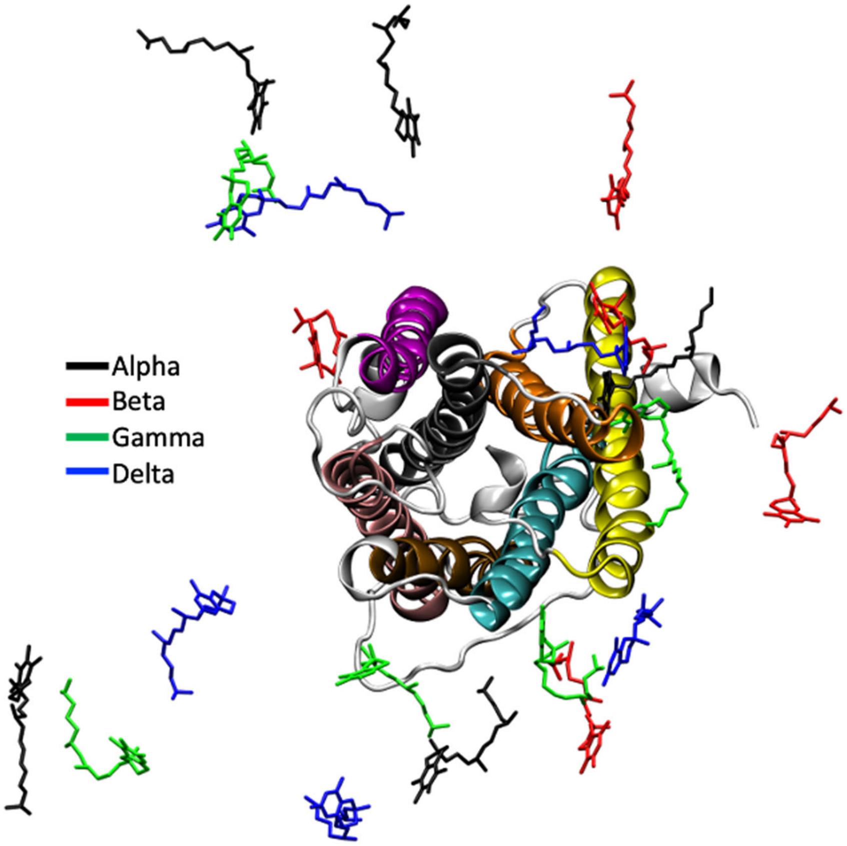

Figure S1. Positions of the tocopherols by the end of simulation.

Table S1. Residues of each transmembrane helices in CB1 and CB2.

Transmembrane Helices	CB2 Residue
H1	29-61
H2	71-97
H3	103-138
H4	147-171
H5	188-220
H6	241-271
H7	276-302

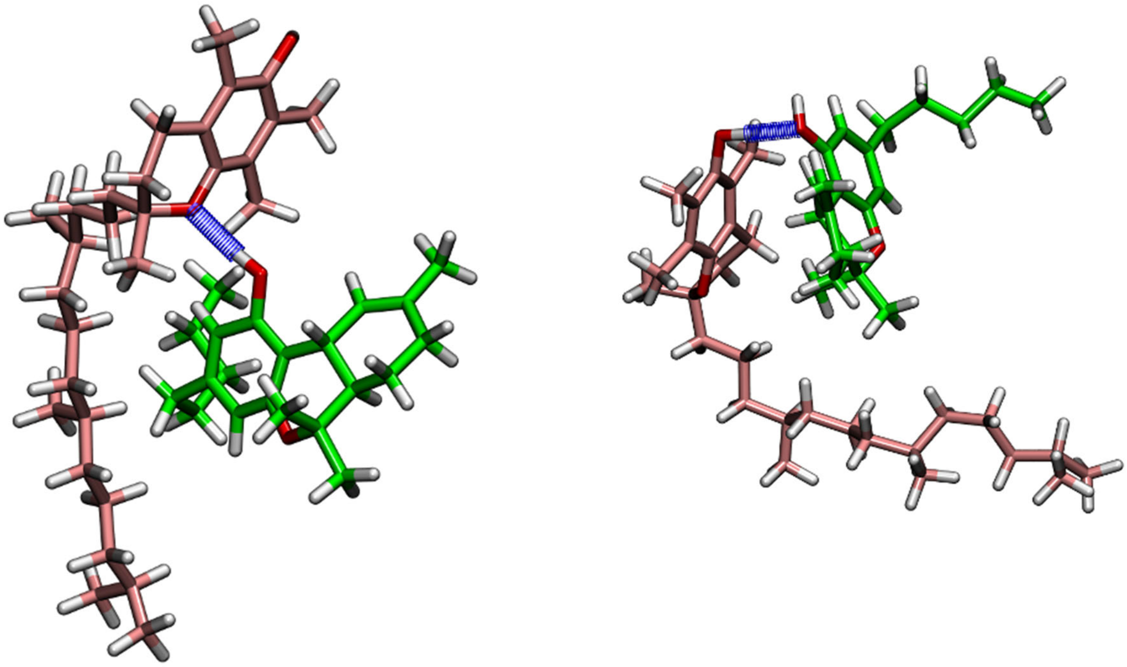

Figure S2. Types of H-bonds between alpha tocopherol (pink) and THC (green).

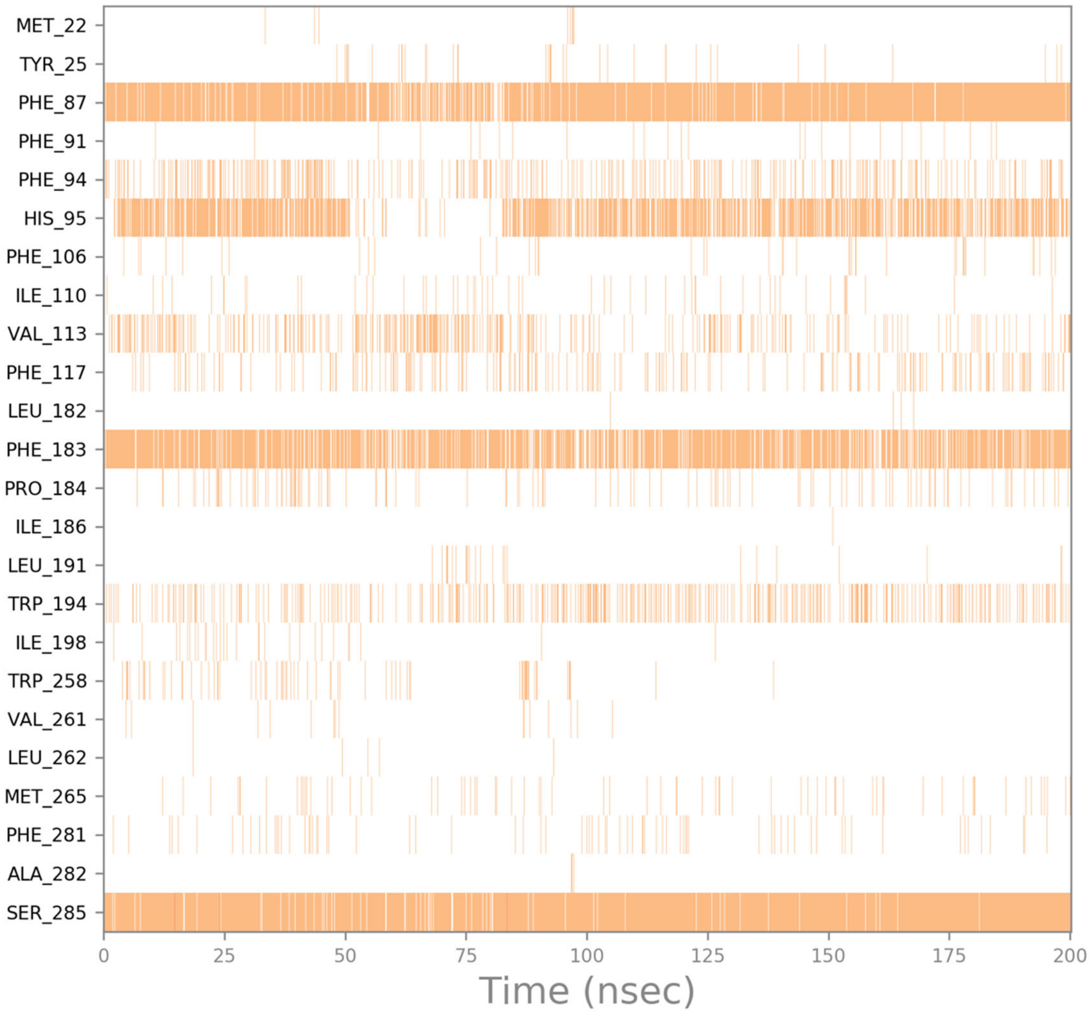

Figure S3. Residues of CB2 interacting with THC during 200ns MD simulation in absence of alpha vitamin E.

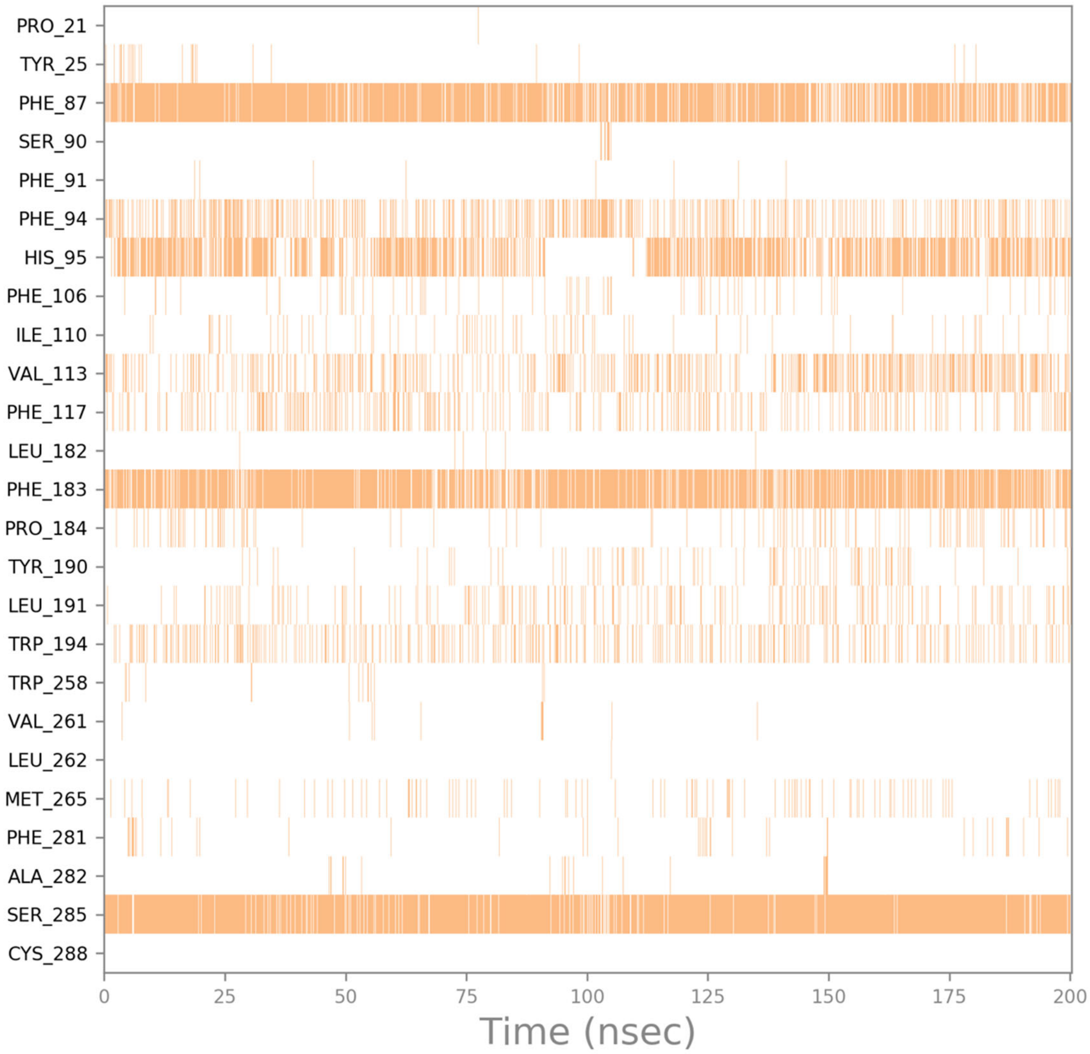

Figure S4. Residues of CB2 interacting with THC during 200ns MD simulation in presence of alpha vitamin E acetate.

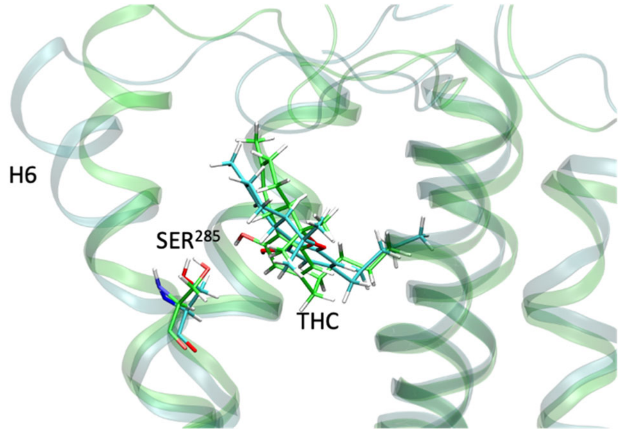

Figure S5. Orientation of THC and SER[285] in presence (cyan color) and absence (green color) of alpha vitamin E around CB2 receptor.
